# Supplementary material for: Optimal age targeting for pneumococcal vaccination in older adults; a modelling study
Source: Nat Commun. 2023 Feb 16;14:888. doi: 10.1038/s41467-023-36624-8 (PMC9935637; doi:10.1038/s41467-023-36624-8)
Supplement: Supplementary file 1 — Supplementary Information [file 41467_2023_36624_MOESM1_ESM.pdf]

Supplementary Appendix

Supplement to: Thindwa D, Clifford S, Kleynhans J, et al. Optimal age targeting for pneumococcal vaccination in older adults; a modelling study.

Table of Contents

Text S1- Description of IPD surveillances in Brazil, England, Malawi and South Africa

Text S2 - Description of the back-inflation method to correct for original IPD cases in England

Text S3 - Description of the exponential growth model fitted to IPD cases data

Table S1 - Comparing preventable IPD cases between age cohorts

Table S2 - Comparing preventable IPD cases under the use of different vaccine products

Table S3 - Comparing preventable IPD cases between fast and slow efficacy waning

Table S4 - Comparing numbers needed to vaccinate to prevent a single case (vaccination efficiency)

Figure S1 - IPD cases in annual age stratified by serotype group, year of surveillance and country

Figure S2 - Reported initial efficacy/effectiveness (VE) and waning VE of pneumococcal vaccines

Figure S3 - Estimated initial VE and waning VE from time since vaccination

Figure S4 - Expected burden of IPD cases across age stratified by serotype group and country

Figure S5 - Age-scaled IPD incidence in age groups stratified by serotype group and country

Figure S6 - The impact of routine pneumococcal vaccination in older adults under the age-dependent initial VE

Figure S7 - The efficiency of routine pneumococcal vaccination in older adults under the age-dependent initial VE

Text S1: Invasive pneumococcal disease surveillance

In Brazil, England, Malawi and South Africa, invasive pneumococcal disease (IPD) in adults is part of their national or subnational disease surveillance. In Brazil, there is population-based surveillance only for meningitis, which are notifiable diseases. For IPD, there is laboratory-based surveillance. The invasive isolates isolated all over the country are sent to the Centre of Bacteriology at Adolfo Lutz Institute (IAL), the Brazilian National Reference Laboratory, for meningitis and IPD characterization. IAL receives IPD isolates collected from private hospitals and laboratories, and from the national network of 25 laboratories coordinated by Brazilian Ministry of Health, with each laboratory covering each Brazilian state [1]. In England, the National Health Service (NHS) hospital laboratories routinely submit IPD isolates to the UK Health Security Agency (UKHSA) for confirmation and serotyping with corresponding electronic reports sent via second generation surveillance system (SGSS) [2]. Reports without isolates are followed-up by UKHSA to request isolate referral to ensure consistently high serotyping rates. In Malawi, an ongoing sentinel surveillance for the laboratory confirmed IPD at a government referral hospital, Queen Elizabeth Central Hospital (QECH), in Blantyre has been in place since 1998, serving now 1.3 million residents of Blantyre district. IPD isolates are submitted to the Malawi Liverpool Wellcome Clinical Research Programme laboratory sitting next to QECH through surveillance programme of IN-patients and Epidemiology system where confirmation and serotyping are conducted [3,4]. In South Africa, the Group for Enteric, Respiratory and Meningeal disease Surveillance in South Africa (GERMS-SA) conducts national, active laboratory-based surveillance across South Africa in a network consisting of nearly 130 public and private microbiology laboratories. Each laboratory submits pneumococcal isolates a long with patient demographics to the Centre for Respiratory Diseases and Meningitis (CRDM) at the National Institute for Communicable Diseases where confirmation and serotyping are performed [5,6].

Text S2: Back-inflation method to correct for IPD cases in England due to the presence of PPV23 vaccination

To estimate the number of English IPD cases assuming absence of PPV23 vaccination in the population, we combined data on PPV23 vaccination coverage (70%), observed number of IPD cases in the presence of PPV23 vaccination program, and age-adjusted vaccine effectiveness of PPV23 of 41% from 0 to <2 years, 34% 2 to <5 years, and 23% from 5+ years as estimated by Djennad et al. The formula below was used to estimate the new number of IPD cases (inflated) assuming the absence of PPV23 vaccination in the older English adult population.

$$\xi_I = \frac{\xi_o}{\nu * (1 - \eta_a) + (1 - \nu)} \dots\dots\dots\dots\dots\dots\dots\dots\dots\dots\dots\dots (1)$$

Where ξ_I is the total IPD cases assuming no PPV23 vaccination in the older English population, ξ_o is the observed total IPD cases in the presence of PPV23 vaccination program, ν is the PPV23 coverage, and η_a is the age (a) adjusted PPV23 vaccine effectiveness from time since vaccination as estimated by Djennad et al.

Text S3: Exponential growth model

To interpolate and extrapolate IPD incidence to annual ages from 55 to 90 years old adults, a nonlinear model (NLS) was fitted to the reported age-specific IPD incidences, minimising the least-squares difference between the model and data using Gauss-Newton algorithm (NLS) as depicted in the main analysis [7]. Initial values of the intercept (α) and gradient (β) for the NLS model were obtained from a fitted linear model, as shown in equation 1.

$$\text{Log}(I|\alpha) = \text{Log}(\gamma) + \beta * \alpha \dots\dots\dots\dots\dots\dots\dots\dots\dots\dots\dots\dots (2)$$

Where I is the IPD incidence for each age group α, γ is the model intercept, and β is the model gradient (growth rate) needed as initial values for equation 2.

Table S1. A base case scenario, age-independent initial vaccine efficacy/effectiveness (VE), using PPV23 under a fast waning VE. Reduction in vaccine-type IPD cases in different vaccination age cohorts (e.g., 55 vs 65 years cohorts) relative to no vaccination scenario.

Country	Vaccination age	2.50%	50%	97.50%
Brazil	55	22.4	30.5	43.3
Brazil	60	20.9	27.3	36.8
Brazil	65	16.8	20.9	26.8
Brazil	70	12.3	15.3	18.9
Brazil	75	8.4	10.4	12.9
Brazil	80	4.9	6.4	8.0
Brazil	85	2.2	2.9	3.8
England	55	6.3	9.2	13.9
England	60	7.7	10.6	15.0
England	65	8.9	11.7	15.6
England	70	10.8	13.7	17.4
England	75	8.7	11.0	13.5
England	80	7.3	9.1	11.1
England	85	4.8	6.0	7.5
Malawi	55	7.7	31.0	96.6
Malawi	60	9.6	30.5	85.9
Malawi	65	9.0	22.4	49.3
Malawi	70	8.2	17.3	35.2
Malawi	75	4.0	7.9	16.2
Malawi	80	2.0	4.6	10.4
Malawi	85	0.9	2.4	6.5
South Africa	55	13.7	20.0	28.7
South Africa	60	13.8	19.2	25.9
South Africa	65	11.9	15.4	20.0
South Africa	70	8.5	10.8	13.7
South Africa	75	6.1	7.7	9.6
South Africa	80	3.8	4.9	6.1
South Africa	85	1.9	2.5	3.2

Table S2. A base case scenario, age-independent initial efficacy/effectiveness (VE), where 55- and 70-years old cohorts are vaccinated under fast waning VE. Reduction in vaccine-type IPD cases between use of different vaccines products (e.g., PCV20 vs PPV23) relative to no vaccination scenario.

Country	Vaccination age	Serotype group	2.50%	50%	97.50%
Brazil	55	PCV13	24.7	38.4	61.5
Brazil	70	PCV13	16.5	21.7	28.7
Brazil	55	PCV15	27.8	43.8	65.0
Brazil	70	PCV15	18.4	23.5	30.3
Brazil	55	PCV20	42.7	61.3	89.2
Brazil	70	PCV20	25.1	32.0	40.4
Brazil	55	PPV23	20.6	30.1	43.5
Brazil	70	PPV23	11.2	15.2	20.0
England	55	PCV13	2.5	4.4	7.7
England	70	PCV13	6.2	8.3	11.1
England	55	PCV15	3.9	6.6	11.0
England	70	PCV15	9.5	12.6	17.0
England	55	PCV20	13.3	19.2	29.2
England	70	PCV20	21.6	27.2	34.5
England	55	PPV23	6.2	9.3	13.7
England	70	PPV23	10.4	13.6	17.6
Malawi	55	PCV13	5.7	30.9	74.0
Malawi	70	PCV13	5.5	16.1	46.0
Malawi	55	PCV15	6.4	32.2	75.3
Malawi	70	PCV15	6.0	17.9	50.0
Malawi	55	PCV20	13.0	64.5	92.2
Malawi	70	PCV20	11.7	37.3	99.0
Malawi	55	PPV23	5.7	28.5	73.3
Malawi	70	PPV23	5.5	15.8	43.1
South Africa	55	PCV13	17.8	26.7	40.8
South Africa	70	PCV13	8.6	11.0	13.9
South Africa	55	PCV15	18.6	29.7	48.7
South Africa	70	PCV15	10.0	12.9	17.2
South Africa	55	PCV20	39.7	53.1	73.2
South Africa	70	PCV20	18.9	23.2	28.4
South Africa	55	PPV23	13.0	19.8	29.2
South Africa	70	PPV23	8.1	10.8	13.8

Table S3. A base case scenario, age-independent initial efficacy/effectiveness (VE), of PPV23 or PCV20 use in 55- and 70- years old cohort. Reduction in vaccine-type IPD cases between fast vs slow waning VE relative to no vaccination scenario.

Country	Serotype group	Waning VE	Vaccination age	2.50%	50%	97.50%
Brazil	PCV20	Fast waning	55	42.7	61.3	89.2
Brazil	PCV20	Slow waning	55	55.0	79.0	94.3
Brazil	PPV23	Fast waning	55	20.6	30.1	43.5
Brazil	PPV23	Slow waning	55	29.0	40.7	57.1
England	PCV20	Fast waning	55	13.3	19.2	29.2
England	PCV20	Slow waning	55	17.1	24.8	37.4
England	PPV23	Fast waning	55	6.2	9.3	13.7
England	PPV23	Slow waning	55	8.3	12.4	18.6
Malawi	PCV20	Fast waning	55	13.0	64.5	83.2
Malawi	PCV20	Slow waning	55	16.7	83.0	96.6
Malawi	PPV23	Fast waning	55	5.7	28.5	73.3
Malawi	PPV23	Slow waning	55	8.0	38.6	77.2
South Africa	PCV20	Fast waning	55	39.7	53.1	73.2
South Africa	PCV20	Slow waning	55	51.2	68.2	93.8
South Africa	PPV23	Fast waning	55	13.0	19.8	29.2
South Africa	PPV23	Slow waning	55	18.2	26.4	38.3
Brazil	PCV20	Fast waning	70	25.1	32.0	40.4
Brazil	PCV20	Slow waning	70	31.2	39.8	50.2
Brazil	PPV23	Fast waning	70	11.2	15.2	20.0
Brazil	PPV23	Slow waning	70	15.3	19.5	25.0
England	PCV20	Fast waning	70	21.6	27.2	34.5
England	PCV20	Slow waning	70	27.0	33.9	42.5
England	PPV23	Fast waning	70	10.4	13.6	17.6
England	PPV23	Slow waning	70	13.8	17.5	22.0
Malawi	PCV20	Fast waning	70	11.7	37.3	99.0
Malawi	PCV20	Slow waning	70	14.5	46.4	72.7
Malawi	PPV23	Fast waning	70	5.5	15.8	43.1
Malawi	PPV23	Slow waning	70	7.5	20.7	54.6
South Africa	PCV20	Fast waning	70	18.9	23.2	28.4
South Africa	PCV20	Slow waning	70	23.4	28.8	35.4
South Africa	PPV23	Fast waning	70	8.1	10.8	13.8
South Africa	PPV23	Slow waning	70	11.0	13.8	17.3

Table S4. A base case scenario, age-independent initial vaccine efficacy/effectiveness (VE), of PPV23 use under fast waning VE. Number needed to vaccinate (NNV) to prevent a single IPD case.

Country	Vaccination age	2.50%	50%	97.50%
Brazil	55	8399	11947	16530
Brazil	60	7868	10461	13934
Brazil	65	8165	10283	13162
Brazil	70	8593	10592	13472
Brazil	75	9210	11271	14171
Brazil	80	10208	12602	16234
Brazil	85	11969	15238	19419
England	55	556	805	1222
England	60	434	594	834
England	65	374	487	647
England	70	336	423	544
England	75	307	378	475
England	80	287	352	438
England	85	292	355	450
Malawi	55	201	738	2835
Malawi	60	244	684	2009
Malawi	65	309	704	1649
Malawi	70	399	759	1552
Malawi	75	469	854	1663
Malawi	80	473	984	2171
Malawi	85	504	1248	3139
South Africa	55	3260	4931	7171
South Africa	60	2909	4083	5589
South Africa	65	2853	3783	4919
South Africa	70	2900	3652	4611
South Africa	75	3004	3643	4555
South Africa	80	3108	3830	4908
South Africa	85	3413	4338	5664

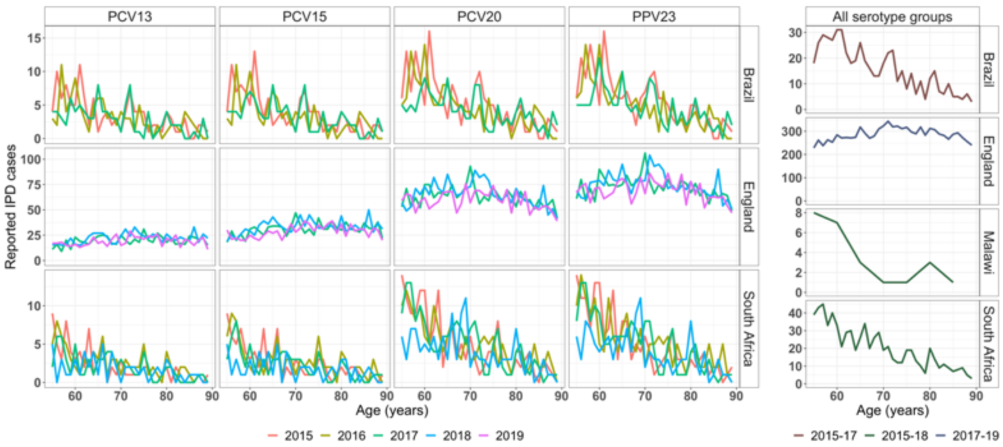

Figure S1. Distribution of invasive pneumococcal disease (IPD) cases in annual age, stratified by serotype group, combined serotype groups, year of surveillance and country. Reported serotype group specific IPD cases by their target pneumococcal vaccines in Brazil (from 2015-2017), England (from 2017-2019) and South Africa (from 2015-2018) in annual age in older adults. Serotype group specific IPD cases in each setting were average for all reported years from 2015 (red), 2016 (olive), 2017 (light green), 2018 (blue) and 2019 (purple) and used to compute serotype group and age specific IPD incidence. Due to small sample size of IPD cases reported and incomplete serotyping data for age-group specific serotype distributions in Malawi, we calculated all-age serotype distribution and assumed the same in each age group. Reported age specific IPD cases for all serotype groups combined for Brazil from 2015-2017 (dark red), England from 2015-2018 (dark blue), Malawi and South Africa from 2017-2019 (dark green)

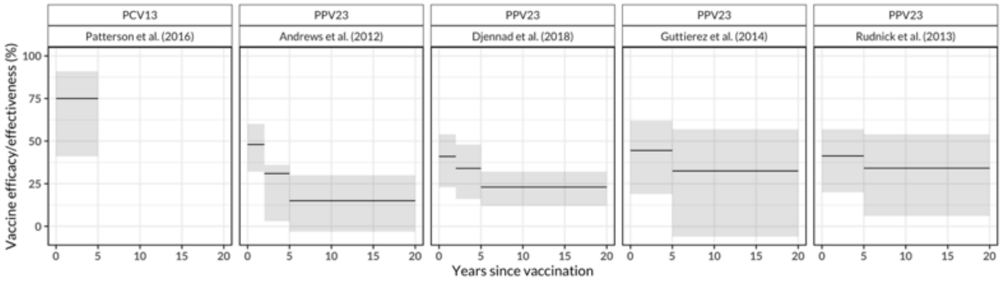

Figure S2. Initial vaccine efficacy/effectiveness (VE) and reported waning VE of pneumococcal vaccines. Reported initial mean VE (black line) and waning VE of pneumococcal polysaccharide vaccine (PPV23) and pneumococcal conjugate vaccine (PCV13), with bootstrapped 95% confidence intervals (gray ribbon) estimated from a piecewise constant model, with time since vaccination and stratification by study's first author name. Initial mean VE and waning VE from Andrews et al. and Djennad et al. are used for the PPV23 in the vaccination impact cohort model as they provide the highest and lowest initial VE levels as well as fast and slow waning VE, respectively, whereas the initial mean VE from Patterson at al. (stable for 5 years) and fast waning VE from Andrews et al. and slow waning VE from Djennad et al. are used for all the PCVs (PCV13, PCV15, and PCV20).

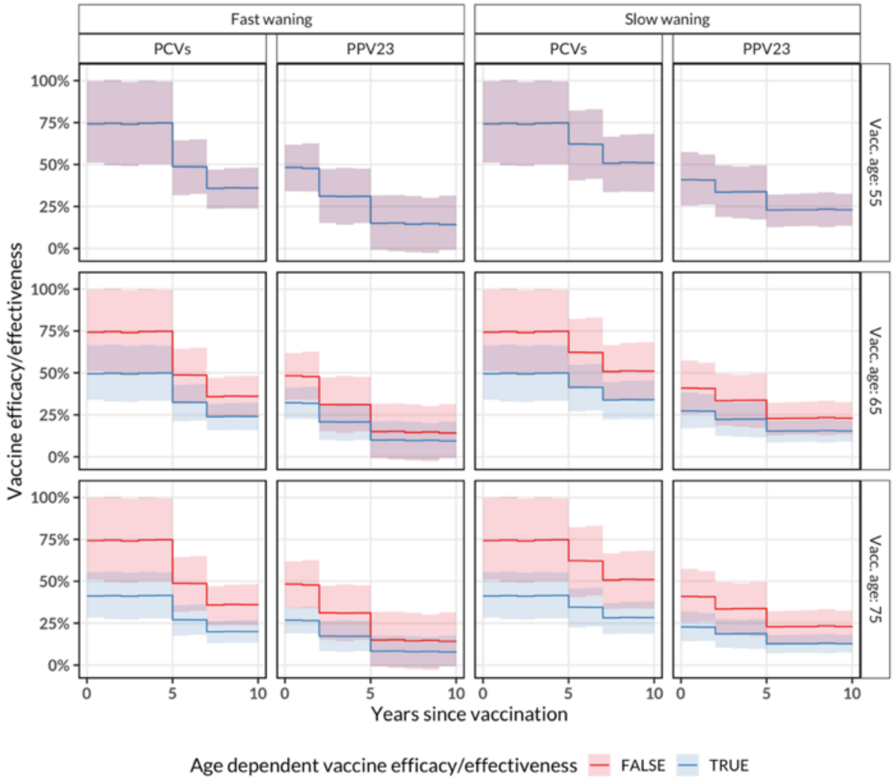

Figure S3. Initial vaccine efficacy/effectiveness (VE) and estimated waning VE of pneumococcal vaccines. Reported age-dependent initial mean VE (blue line) and age-independent initial mean VE (red line) and estimated waning VE of pneumococcal polysaccharide vaccine (PPV23) and pneumococcal conjugate vaccine (PCV13) with respective bootstrapped 95% confidence intervals (ribbons), estimated from a piecewise constant model, with time since vaccination and stratification by study's first author name and *a priori* age of 65 and 75 years old at which initial VE is lower than 55 years old.

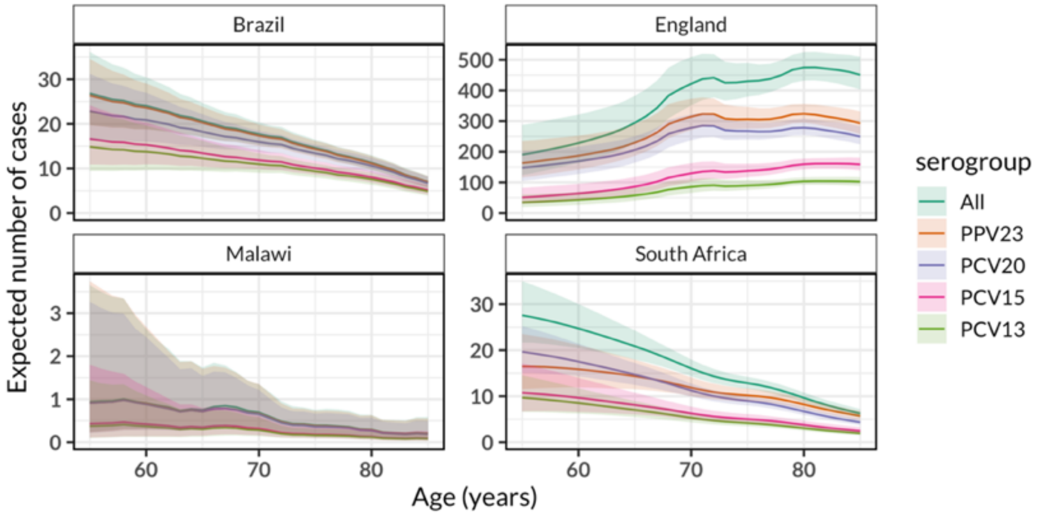

Figure S4. The expected burden of invasive pneumococcal disease (IPD) cases by age, serogroup and country. The expected burden of IPD cases across age stratified by serotype group in Brazil (from 2015-2017), England (from 2017-2019) and South Africa (from 2015-2018) in annual age in older adults. Serotype group specific expected number of IPD cases (lines) in each setting was obtained by computing the product of observed IPD cases aggregated for all reported years and the fitted IPD incidence. The 95% confidence intervals in the expected number of cases (ribbon) were based on uncertainty in the fitted incidence obtained by bootstrap sampling 1000 times using the fitted parameter means and covariance matrix of a fitted exponential model.

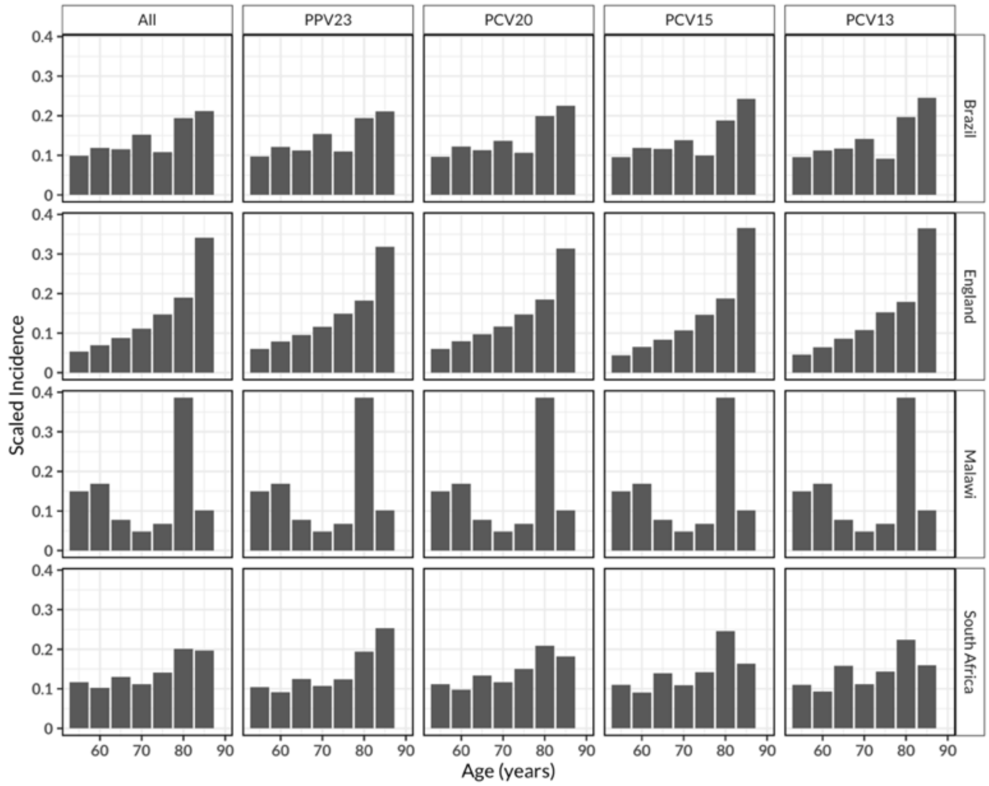

Figure S5. Age-scaled invasive pneumococcal disease (IPD) incidence in age groups stratified by serotype group and country for all years of IPD surveillance in a mature infant PCV era (at least four years post-PCV introduction). Age-scaled IPD incidence is estimated by dividing age group-specific IPD incidence by total incidence across all age groups within that serotype group. Age-scaled IPD incidence increases monotonically with increasing age irrespective of serotype group or country except for Malawi where the unstable signal in age-scaled IPD incidence reflects small numbers of reported IPD cases.

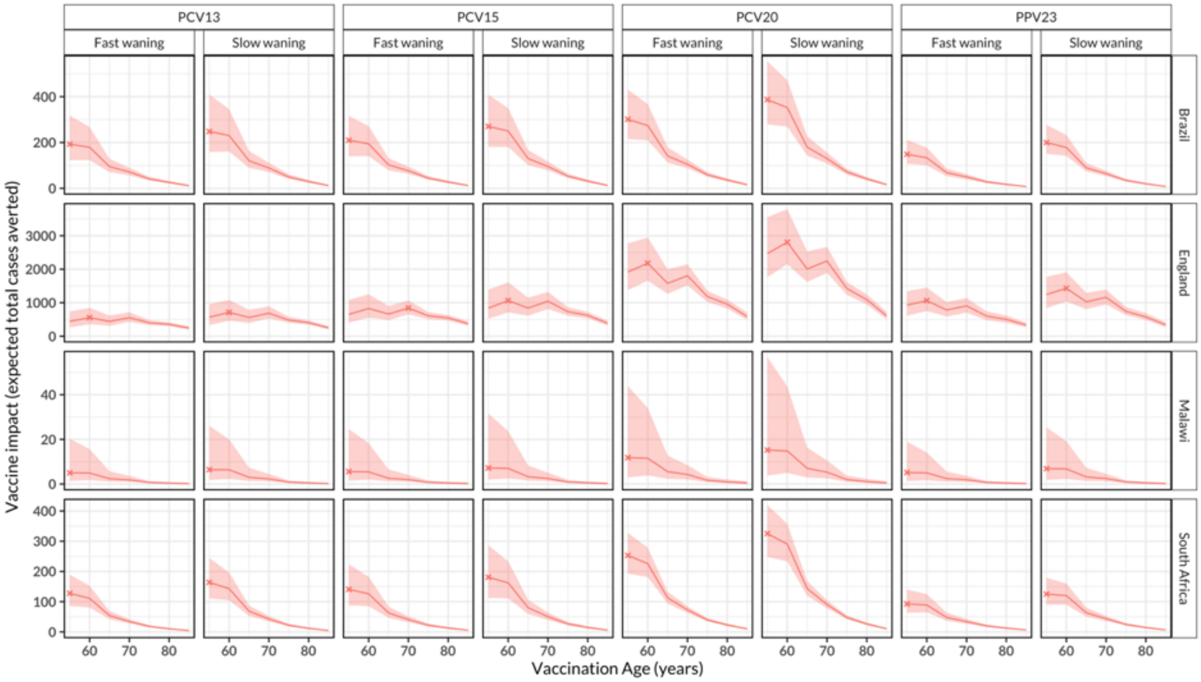

Figure S6. The impact of routine pneumococcal vaccination in older adults aged ≥55 years old (y). The expected absolute number of total IPD cases averted for the rest of age cohort lifetime by vaccinating every older adult in the age cohort stratified by country and vaccine product, under alternative scenario of age-dependent initial vaccine efficacy/effectiveness (VE) and waning VE in Brazil, England, Malawi and South Africa. The red lines represent cohort model mean estimates, and the shaded red ribbon represents 95% bootstrap confidence intervals for the mean estimates. The X represents the optimal age for pneumococcal vaccination. In Brazil, Malawi and South Africa, most cases are prevented at age 55y whereas in England this is achieved mostly at age 60y.

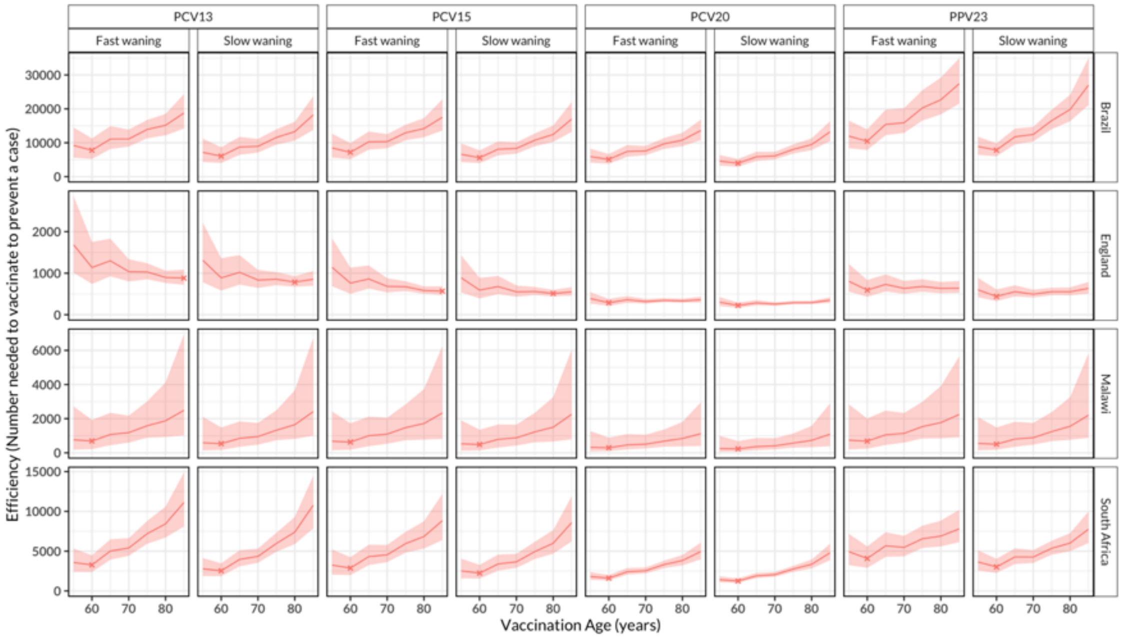

Figure S7. The efficiency of routine pneumococcal vaccination in older adults aged ≥55 years old (y). The number of individuals who are needed to vaccinate in each age of vaccination to prevent a case, stratified by country and vaccine product, under alternative scenario of age-dependent initial vaccine efficacy/effectiveness (VE) and waning VE in Brazil, England, Malawi and South Africa. The red lines represent cohort model mean estimates, and the shaded red ribbon represents 95% bootstrap confidence intervals for the mean estimates. The X represents the optimal age for efficiency of pneumococcal vaccination program. In Brazil, Malawi and South Africa, optimal age of vaccination efficiency is achieved in 60y age cohort irrespective of vaccine product or waning VE assumption whereas in England, efficiency is achieved in 60y age cohort for PCV20 and PPV23 irrespective of waning VE assumption, and in 80y or 85y for PCV13 and PCV15's slow or fast waning VE, respectively.

References

[1] Brandileone M-CC, Almeida SCG, Minamisava R, Andrade A-L. Distribution of invasive Streptococcus pneumoniae serotypes before and 5 years after the introduction of 10-valent pneumococcal conjugate vaccine in Brazil. Vaccine 2018;36:2559–66. https://doi.org/10.1016/j.vaccine.2018.04.010.

[2] Djennad A, Ramsay ME, Pebody R, Fry NK, Sheppard C, Ladhani SN, et al. Effectiveness of 23-Valent Polysaccharide Pneumococcal Vaccine and Changes in Invasive Pneumococcal Disease Incidence from 2000 to 2017 in Those Aged 65 and Over in England and Wales. EClinicalMedicine 2019;6:42–50. https://doi.org/10.1016/j.eclinm.2018.12.007.

[3] Bar-Zeev N, Swarthout TD, Everett DB, Alaerts M, Msefula J, Brown C, et al. Impact and effectiveness of 13-valent pneumococcal conjugate vaccine on population incidence of vaccine and non-vaccine serotype invasive pneumococcal disease in Blantyre, Malawi, 2006–18: prospective observational time-series and case-control studies. Lancet Glob Health 2021;9:e989–98. https://doi.org/10.1016/S2214-109X(21)00165-0.

[4] SanJoaquin MA, Allain TJ, Molyneux ME, Benjamin L, Everett DB, Gadabu O, et al. Surveillance Programme of IN-patients and Epidemiology (SPINE): Implementation of an Electronic Data Collection Tool within a Large Hospital in Malawi. PLOS Med 2013;10:e1001400. https://doi.org/10.1371/journal.pmed.1001400.

[5] Meiring S, Cohen C, Quan V, Gouveia L de, Feldman C, Karstaedt A, et al. HIV Infection and the Epidemiology of Invasive Pneumococcal Disease (IPD) in South African Adults and Older Children Prior to the Introduction of a Pneumococcal Conjugate Vaccine (PCV). PLOS ONE 2016;11:e0149104. https://doi.org/10.1371/journal.pone.0149104.

[6] Kleynhans J, Cohen C, McMorrow M, Tempia S, Crowther-Gibson P, Quan V, et al. Can pneumococcal meningitis surveillance be used to assess the impact of pneumococcal conjugate vaccine on total invasive pneumococcal disease? A case-study from South Africa, 2005–2016. Vaccine 2019;37:5724–30. https://doi.org/10.1016/j.vaccine.2019.04.090.

[7] Srinivasan V, Mason CH. Nonlinear Least Squares Estimation of New Product Diffusion Models. Mark Sci 1986;5:169–78.
