# Supplementary material for: Optimal age targeting for pneumococcal vaccination in older adults; a modelling study
Source: Nat Commun. 2023 Feb 16;14:888. doi: 10.1038/s41467-023-36624-8 (PMC9935637; doi:10.1038/s41467-023-36624-8)
Supplement: Supplementary file 3 — Reporting Summary [file 41467_2023_36624_MOESM3_ESM.pdf]

nature portfolio

Corresponding author(s): Deus Thindwa

Last updated by author(s): 2023/02/04

Reporting Summary

Nature Portfolio wishes to improve the reproducibility of the work that we publish. This form provides structure for consistency and transparency in reporting. For further information on Nature Portfolio policies, see our Editorial Policies and the Editorial Policy Checklist.

Statistics

For all statistical analyses, confirm that the following items are present in the figure legend, table legend, main text, or Methods section.

n/a	Confirmed	
☐	☒	The exact sample size (*n*) for each experimental group/condition, given as a discrete number and unit of measurement
☐	☒	A statement on whether measurements were taken from distinct samples or whether the same sample was measured repeatedly
☐	☒	The statistical test(s) used AND whether they are one- or two-sided<br>*Only common tests should be described solely by name; describe more complex techniques in the Methods section.*
☐	☒	A description of all covariates tested
☒	☐	A description of any assumptions or corrections, such as tests of normality and adjustment for multiple comparisons
☐	☒	A full description of the statistical parameters including central tendency (e.g. means) or other basic estimates (e.g. regression coefficient) AND variation (e.g. standard deviation) or associated estimates of uncertainty (e.g. confidence intervals)
☒	☐	For null hypothesis testing, the test statistic (e.g. *F*, *t*, *r*) with confidence intervals, effect sizes, degrees of freedom and *P* value noted<br>*Give P values as exact values whenever suitable.*
☒	☐	For Bayesian analysis, information on the choice of priors and Markov chain Monte Carlo settings
☒	☐	For hierarchical and complex designs, identification of the appropriate level for tests and full reporting of outcomes
☒	☐	Estimates of effect sizes (e.g. Cohen's *d*, Pearson's *r*), indicating how they were calculated

Our web collection on statistics for biologists contains articles on many of the points above.

Software and code

Policy information about availability of computer code

Data collection	Study data were collected and shared via Microsoft Excel 2016 and Microsoft Access 2016; IPD data were collected and shared by Institutional pneumococcal disease laboratories in the four countries whereas country demographics data were scraped from national census websites and reports. Vaccine efficacy/effectiveness data were extracted from the published literature from six studies in Europe.
Data analysis	A Gauss-Newton algorithm (implemented by nls function from stats base R package) was used to minimise the least-squares difference between the model and data during nonlinear model (NLS) fitted to the reported age-specific IPD incidences. All analyses were conducted using R language v4·1·1, with code freely availabile through Zenodo; https://doi.org/10.5281/zenodo.7592905

For manuscripts utilizing custom algorithms or software that are central to the research but not yet described in published literature, software must be made available to editors and reviewers. We strongly encourage code deposition in a community repository (e.g. GitHub). See the Nature Portfolio guidelines for submitting code & software for further information.

Data

Policy information about availability of data

All manuscripts must include a data availability statement. This statement should provide the following information, where applicable:

- Accession codes, unique identifiers, or web links for publicly available datasets
- A description of any restrictions on data availability
- For clinical datasets or third party data, please ensure that the statement adheres to our policy

IPD surveillance, country demographics and vaccine efficacy/effectiveness data were used in this study. Aggregate and anonymised full data used in this study are freely available in the Zenodo database hosted at; https://doi.org/10.5281/zenodo.7592905

Human research participants

Policy information about studies involving human research participants and Sex and Gender in Research.

Reporting on sex and gender	This information was not relevant for this analysis that looked at optimal age of vaccination for older adults with pneumococcal vaccines.
Population characteristics	Covariates in this study only included age and HIV status
Recruitment	Participants were IPD patients showing up at clinics/hospitals in different countries. In Brazil, England and South Africa, these are national-wide IPD surveillance programmes whereas in Malawi, data were from Blantyre only (sub-national) thereby may not give a representation of the entire country.
Ethics oversight	The London School of Hygiene and Tropical Medicine Research Ethics Committee (25787).

Note that full information on the approval of the study protocol must also be provided in the manuscript.

Field-specific reporting

Please select the one below that is the best fit for your research. If you are not sure, read the appropriate sections before making your selection.

☒ Life sciences ☐ Behavioural & social sciences ☐ Ecological, evolutionary & environmental sciences

For a reference copy of the document with all sections, see nature.com/documents/nr-reporting-summary-flat.pdf

Life sciences study design

All studies must disclose on these points even when the disclosure is negative.

Sample size	No sample size calculation was undertaken as we used all the data, from the pneumococcal surveillance programmes over the years post-infant pneumococcal vaccination programmes in Brazil, England and South Africa, which were large enough. However, data from Malawi were not large enough due to resource-limitations and we made assumptions about having similar pneumococcal serotype group distribution in each age group as across all ages, which may potentially result in biases if serotype group distribution is distinct in each age group. This weakness has also been included in the manuscript "Discussion" section.
Data exclusions	In this analysis, we largely focused on older adults living without HIV. Thus, in South Africa and Malawi where HIV prevalence is relatively high, we adjusted for the proportion of IPD cases with HIV.
Replication	We have developed analysis scripts which are publicly shared in the Github repository. We have rerun the scripts several times to yield the same results (reproducibility) (https://github.com/deusthindwa/optimal.age.targeting.pneumo.vaccines).
Randomization	Randomization was not relevant for this study as the nature of the study design is not suited for randomization.
Blinding	Investigators were naturally blinded at different stages e.g. lab technician performing sampling and pneumococcal detection have never discussed lab experiments and results with researchers performing statistical analyses. Moreover, our investigation in this manuscript was not known in advance during or prior to data collection and sample processing (it is a retrospective data analysis).

Reporting for specific materials, systems and methods

We require information from authors about some types of materials, experimental systems and methods used in many studies. Here, indicate whether each material, system or method listed is relevant to your study. If you are not sure if a list item applies to your research, read the appropriate section before selecting a response.

Materials & experimental systems

n/a	Involved in the study
☒	☐ Antibodies
☒	☐ Eukaryotic cell lines
☒	☐ Palaeontology and archaeology
☒	☐ Animals and other organisms
☐	☒ Clinical data
☒	☐ Dual use research of concern

Methods

n/a	Involved in the study
☒	☐ ChIP-seq
☒	☐ Flow cytometry
☒	☐ MRI-based neuroimaging

Clinical data

Policy information about clinical studies

All manuscripts should comply with the ICMJE guidelines for publication of clinical research and a completed CONSORT checklist must be included with all submissions.

Clinical trial registration: Not applicable

Study protocol: Protocols for the national and sub-national invasive pneumococcal surveillance programmes are publicly available from the health departments of each country, or links can also be requested from the first author if needed.

Data collection: In Brazil, England, Malawi and South Africa, invasive pneumococcal disease (IPD) in adults is part of their national or subnational disease surveillance. In Brazil, there is population-based surveillance only for meningitis, which are notifiable diseases. For IPD, there is laboratory-based surveillance. The invasive isolates isolated all over the country are sent to the Centre of Bacteriology at Adolfo Lutz Institute (IAL), the Brazilian National Reference Laboratory, for meningitis and IPD characterization. IAL receives IPD isolates collected from private hospitals and laboratories, and from the national network of 25 laboratories coordinated by Brazilian Ministry of Health, with each laboratory covering each Brazilian state. In England, the National Health Service (NHS) hospital laboratories routinely submit IPD isolates to the UK Health Security Agency (UKHSA) for confirmation and serotyping with corresponding electronic reports sent via second generation surveillance system (SGSS). Reports without isolates are followed-up by UKHSA to request isolate referral to ensure consistently high serotyping rates. In Malawi, an ongoing sentinel surveillance for the laboratory confirmed IPD at a government referral hospital, Queen Elizabeth Central Hospital (QECH), in Blantyre has been in place since 1998, serving now 1.3 million residents of Blantyre district. IPD isolates are submitted to the Malawi Liverpool Wellcome Clinical Research Programme laboratory sitting next to QECH through surveillance programme of IN-patients and Epidemiology system where confirmation and serotyping are conducted. In South Africa, the Group for Enteric, Respiratory and Meningeal disease Surveillance in South Africa (GERMS-SA) conducts national, active laboratory-based surveillance across South Africa in a network consisting of nearly 130 public and private microbiology laboratories. Each laboratory submits pneumococcal isolates a long with patient demographics to the Centre for Respiratory Diseases and Meningitis (CRDM) at the National Institute for Communicable Diseases where confirmation and serotyping are performed.

Outcomes: Our disease endpoint was invasive pneumococcal disease, with outcome being age of vaccination of a single dose pneumococcal vaccine to prevent maximum invasive pneumococcal disease cases. Vaccination impact for vaccinating age cohort was assessed by combining vaccine efficacy/effectiveness estimates and expected number of invasive pneumococcal disease cases (driven by invasive pneumococcal disease incidence and smoothed population demographics for each country) in a cohort model.
